# Effect of occupational mobility and health status on life satisfaction of Chinese residents of different occupations: logistic diagonal mobility models analysis of cross-sectional data on eight Chinese provinces

**DOI:** 10.1186/1475-9276-13-15

**Published:** 2014-02-08

**Authors:** Ying Liang, Peiyi Lu

**Affiliations:** 1 Department of Social Work and Social Policy, School of Social and Behavioral Sciences, Nanjing University, Nanjing 210023, People’s Republic of China; 2 School of Communication and Design, Sun Yat-sen University, Guangzhou, People’s Republic of China

**Keywords:** Occupational mobility, Life satisfaction, Health status, Chinese residents of different occupations, Logistic DMMs

## Abstract

**Background:**

Life satisfaction research in China is in development, requiring new perspectives for enrichment. In China, occupational mobility is accompanied by changes in economic liberalization and the emergence of occupational stratification. On the whole, however, occupational mobility has rarely been used as an independent variable. Health status is always used as the observed or dependent variable in studies of the phenomenon and its influencing factors. A research gap still exists for enriching this field.

**Methods:**

The data used in this study were obtained from the China Health and Nutrition Survey (CHNS). The study included nine provinces in China. The survey was conducted from 1989 to 2009.Every survey involved approximately 4400 families or 19,000 individual samples and parts of community data.

**Results:**

***First,*** we built a 5 × 5 social mobility table and calculated life satisfaction of Chinese residents of different occupations in each table. ***Second,*** gender, age, marital status, education level, annual income and hukou, health status, occupational mobility were used as independent variables. ***Lastly,*** we used logistic diagonal mobility models to analyze the relationship between life satisfaction and the variables. Model 1 was the basic model, which consisted of the standard model and controlled variables and excluded drift variables. Model 2 was the total model, which consisted of all variables of interest in this study. Model 3 was the screening model, which excluded the insignificant drift effect index in Model 2.

**Conclusion:**

From the perspective of the analysis of controlled variables, health conditions, direction, and distance of occupational mobility significantly affected life satisfaction of Chinese residents of different occupations. (1) From the perspective of health status, respondents who have not been sick or injured had better life satisfaction than those who had been sick or injured. (2) From the perspective of occupational mobility direction, the coefficients of occupational mobility in the models are less than 0, which means that upward mobility negatively affects life satisfaction. (3) From the perspective of distance, when analyzing mobility distance in Models 2 and 3, a greater distance indicates better life satisfaction.

## Introduction

### Life satisfaction and its influencing factors

As an important index of the quality of human life, cognition, and emotion, life satisfaction is often discussed in countries experiencing a transition period. The concept of life satisfaction stems from subjective well-being, people’s cognitive evaluation of their life [1] and the subjective overall rating of individuals based on their own defining criteria. Life satisfaction is also called subjective quality of life, society members’ evaluation of their various environments. Life satisfaction, along with the creation of prevention and intervention programs, is a discipline that aims to improve the quality of life of all individuals [2].

Life satisfaction can generally be measured in two ways: through a one-dimensional model or through a multidimensional model. A one-dimensional model is used to measure overall satisfaction in life. The theoretical assumption is that individuals assess their life satisfaction based on their perception of general life; this has nothing to do with special life satisfaction [3]. A typical one-dimensional model is a single questionnaire that asks respondents about their overall life satisfaction. This measurement is commonly used in large-scale, nationwide surveys, even in international surveys [4-7]. A multidimensional model can measure both specific life satisfaction and overall life satisfaction. This model is commonly used in small-scale sample surveys and involves complex scoring. There are many pros and cons with this model; hence, consensus regarding its use in psychology has not been reached [3].

Most studies of life satisfaction have focused on its influencing factors. The best predictors are socio-economic status and perceived health status [8]. Meanwhile, many other studies have focused on the effect of different groups’ socio-economic status on life satisfaction. Economic satisfaction and life satisfaction are strongly associated in poor countries, whereas home life satisfaction is strongly associated with life satisfaction in rich countries [9]. Self-rated health is a predominant variable of life satisfaction and most explained variables [10]. Few studies have explored the relationship between health and life satisfaction, especially among the Chinese residents of different occupations.

Studies of life satisfaction are common in China, and research perspectives on this subject are diverse. ***First,*** many studies of different groups have been conducted. Many groups have focused on old people [11-14], whereas several studies have focused on children and adolescents, such as AIDS orphans [15] and children of migrant workers in Shanghai [16]. Several other studies have investigated special groups, such as research and development scientists [17]. ***Second,*** many studies have focused on factors of Chinese life satisfaction. Perceived fairness affects Chinese people’s evaluation of life satisfaction [4]. For Chinese peasants, communist party membership and political participation can improve life satisfaction. Economic growth and low inflation can also improve life satisfaction [6]. ***Third,*** some studies have focused on the reasons for low life satisfaction. Some others have described Chinese life satisfaction between 1990 and 2010 and find that similar to Central and Eastern Europe, China is in a track transition: a U-shaped swing and a zero or downward trend. This transition can be attributed in part to the growing income inequality [18]. These observations indicate that life satisfaction research in China is in development, requiring new perspectives for enrichment.

### Occupational mobility in Chinese society

With the rapid economic development of China, Chinese society faces a complex period of transition. Under the occupational stratification background, occupational mobility has gained considerable attention. In 2010, the urbanization level was at 46.6%, an increase of 28.7 percentage points from that in 1978. In 2011, the total migrant population of China was approximately 230 million, accounting for 17% of the total population of the country [19]. China’s social structure is changing. A prominent aspect of this change is huge demographic changes in the occupational structure. Business and politics engaged in the management and service occupations have increased significantly, and many farmers and workers have changed their careers [20].

With the lessons China has learned about the reform boom, research about occupational mobility has also become very popular. China initially formed a model of the modern mechanism of social mobility. This mechanism for a diversified and open channel of social mobility is an important factor. Social mobility has opened gradually in the post-economic reform era, as seen in rural residents coming to towns for work, urban citizens becoming self-employed or having private businesses, and colleges and universities restoring their enrollment. Therefore, the institutional arrangements and policies defining the social status patterns of the people have been directly broken.

Occupational mobility refers to the change in position of people in the social professional hierarchy. Reasonable occupational mobility is a necessary condition for social development and an important coordination mechanism for a functioning society. Work is an important part of human life [21]. Sociology considers social status to depend on three factors: power, wealth, and prestige. Wealth and prestige are difficult to operate in empirical studies. Career reflects power, wealth, and prestige to a large extent in modern society [20]. Why then do people shift to a different career? Occupational mobility occurs when employees want a higher pay or the company hopes to have a more efficient staff [22]. As rational individuals, most workers change jobs to achieve upward mobility and higher economic and non-economic compensation packages (such as a superior work environment and better promotion prospects) [23]. Occupation is a good indicator of social position, and a long-standing research tradition in sociology documents how to construct measures of rank and class using occupational information.

In China, occupational mobility is accompanied by changes in economic liberalization and the emergence of social mobility. Under the conditions of the post-1970s Chinese market economy, the huge transformation of social institutions and structure in China led to large-scale social mobility and occupational stratification. In 1959, China introduced the household registration system, the Hukou, which restricted citizens to a fixed city or a rural area. Rural residents were prohibited from city, inter-firm, or inter-sector mobility, which was extremely difficult before the implementation of reforms [24,25]. Since the economic reform of the 1970s, China has undergone tremendous transformation in society, economy, and cultural structures [26]. In the resumption of the financial position of China, the rapid economic development in the city created gaps between urban and rural areas [27,28]. This development led many rural residents to the cities to look for work and a better life. Thus, China underwent the largest scale of social mobility in the late 1970s.

Occupational mobility studies in China have matured. ***First,*** scholars not only have studied the basic conditions, characteristics, and factors of occupational mobility as a whole but have also focused on the two important indicators of intergenerational and intragenerational mobility, which reflect the level and status of social development. In addition, the occupational mobility of migrant workers in the city has become a focus of academic attention [29]. System changes have affected the overall pattern of occupational mobility and the main factors in the attainment of the elite of occupational status [30]. The evolving political and economic institutions in China have conceivably created uncertainties and unpredictable patterns through research for status attainment, career mobility into elite groups, and social network approaches to occupational processes [31].

***Second,*** many studies have focused on the socio-economic aspects and causes of occupational mobility. Family characteristics, social capital, and labor market structure changes significantly affect gender differences in career mobility [32]. A scholar compared groups with high and low levels of education in terms of occupational mobility pattern and income differences and found that occupational mobility increased among workers with high levels of education [33]. A substantial increase in labor supply may inhibit the tendency of the low-end labor market occupational mobility of workers. In addition, the Chinese occupational mobility ratio has yet to reach the level of developed countries 30 years ago, which was quite low. These findings indicate that there are barriers to occupational mobility. Such barriers can be divided into personal and institutional barriers and barriers to entry and exit [34].

***Third,*** occupational mobility is sometimes used as an independent variable to explore its effect on certain economic variables. For low-education workers, occupational mobility is the most important factor to improve their income levels. By contrast, for highly educated workers, occupational mobility has no effect on income, and the most important factor affecting their income stratification is human capital [35]. On the whole, however, occupational mobility has rarely been used as an independent variable, to study its impact on those social psychology variables.

### Health status of Chinese residents of different occupations

Given that China is the second largest economy in the world, the health of the Chinese is a popular research topic. Research perspectives are also diverse. ***First,*** many studies have focused on the incidence of specific diseases [36], such as chronic kidney disease [37] and diabetes [38]. Some studies have focused on the control of diseases, especially of schistosomiasis [39,40], and of smoking [41,42]. Overall, the development of health status in China still requires improvement. For example, resources and sustainability are important issues [43]. ***Second,*** many studies have focused on giving explanations and suggestions for Chinese health. The physical activity of adults has declined in the past 15 years because of higher educational institutions and housing infrastructure, etc. [44]. Researchers suggested primary health care [45], emphasizing the importance of the spread of health literacy [46]. ***Third,*** with improving environmental awareness, many studies have focused on the effect of environmental damage on public health. In China, rapid economic development has brought environmental problems, especially water and air pollution, which has affected public health [47-49].

For Chinese residents of different occupations, using health as an observed or dependent variable to study the phenomenon and its influencing factors is the most common research idea. Health inequality is a popular topic. Many scholars believe that the situation in China is serious [50]. Gaps exist in the region and between social groups [51]. Many studies have investigated the health inequalities between rural and urban areas and have found that the gap is increasing [52,53]. In addition, factors that affect workers’ health have been discussed in great detail. In China, many people from the countryside go to the cities to work. Because of the institutional barrier, the city’s welfare and facilities do not benefit all residents of different occupations. Workers have a higher prevalence of depression symptoms than the general population. Self-rated health and socio-economic status negatively affect depression [54]. The huge gap between low income and high medical costs significantly prevents health-seeking behavior [55].

These findings draw our attention and reflection. Few studies have used the health status of Chinese residents of different occupations as an independent variable to study its effect on other variables. This is a very important and integral part of health research. A 20-year follow-up study found that baseline life satisfaction is related to high-risk suicide, which is partly mediated by poor health behavior [56]. Thus, the health status of the effect mechanism of other variables, such as sociology, psychology, and economics, needs to be studied for greater perspective.

### Research questions, research hypotheses and research objectives

The review of literature revealed that life satisfaction, occupational mobility, and health in China are in development and are constantly improving. China is undergoing a new round of economic and social reform, and occupational stratification is rampant. In this background, discussing these issues is very meaningful. We are wondering whether these three issues have certain relationships. The dependent variable in this article is the life satisfaction of Chinese residents of different occupations, then how are they under different sociological population control variables statistically? Thus, we propose our first hypothesis.

Hypothesis 1: There are significant differences between the life satisfactions of Chinese residents of different occupations under different control variables.

Although the health of Chinese residents of different occupations has been discussed in depth, few studies have used the health of Chinese residents of different occupations as an independent variable to study its effect on other variables. In the background of the growing emphasis on life satisfaction, we hypothesize that the situation can improve a person’s life satisfaction and vice versa. These issues are what we discuss and try to resolve. Thus, we propose our second hypothesis.

Hypothesis 2: Health status has impact on the life satisfaction of Chinese residents of different occupations.

China began its reform in the late 1970s with the set-up of the market economy. This reform led not only to large-scale social mobility but also to significant occupational mobility. As a normal social phenomenon under these conditions, occupational mobility developed based on a combination of social and individual factors. For a person’s life and social development, occupational mobility produces a crucial effect. Are there any relationships on their evaluation of life satisfaction? If so, what is the mechanism? Thus, we propose our third hypothesis.

Hypothesis 3: Occupational mobility has impact on the life satisfaction of Chinese residents of different occupations. And the impact mechanism is complex.

This study used an open database for the health and living conditions of Chinese residents of different occupations. First, we used a logistic diagonal mobility model to explore the effect of the diagonal flow of the sociological and demographic variables of Chinese life satisfaction. Second, we used decade tracking data to test the effects of occupational mobility on people’s life satisfaction. We study the effect of occupational mobility on people’s life satisfaction with the DMM models. We then compared the explanatory power of social choice theory and life satisfaction by calculating the change in different sectors of life satisfaction after the occupational mobility gradient. Finally, we analyzed the effect of health status on life satisfaction. This study aimed to (1) provide the life satisfaction differences in Chinese residents of different occupations in terms of gender, age, education, and other basic sociological demographic variables and to improve life satisfaction research and development in China; to (2) investigate occupational mobility and the health effect mechanism of life satisfaction of Chinese residents of different occupations and gain an in-depth understanding of the life satisfaction factors of residents of different occupations; and to (3) provide advice on the life satisfaction of different occupations from the perspective of occupational mobility and health.

## Methods

### Data resources

The data used in this study were obtained from the China Health and Nutrition Survey (CHNS)^a^, which was conducted by North Carolina University and the Chinese Center for Disease Control and Prevention. The study included nine provinces (Liaoning, Heilongjiang, Jiangsu, Shandong, Henan, Hubei, Hunan, Guangxi, and Guizhou) in China, which are different in terms of geographic characteristics, economic development level, public resources, and health index. The surveys were conducted in 1989, 1991, 1993, 1997, 2000, 2004, 2006, and 2009. Every survey involved approximately 4400 families or 19000 individual samples and parts of community data. This survey used multi-stages and random clustering methods, where the variables included were gender, education level, marital status, and income. The representativeness of the sample was relatively fair.

**Figure 1:**
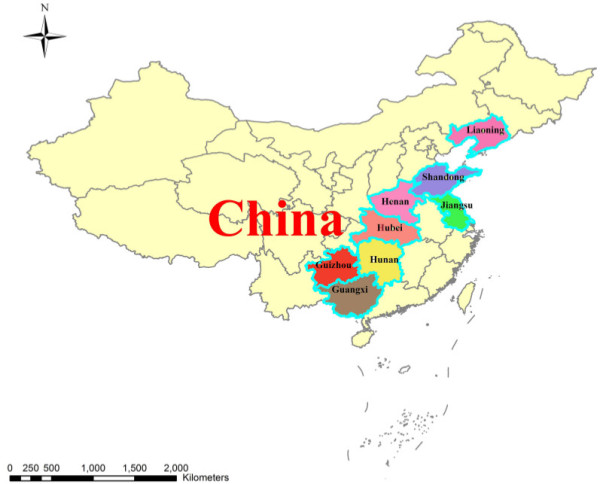
Eight provinces involved in the CHNS survey.

The subject provinces in 1989, 1991, and 1993 were Liaoning, Jiangsu, Shandong, Henan, Hubei, Hunan, Guangxi, and Guizhou. In 1997, the subjects were Heilongjiang, Jiangsu, Shandong, Henan, Hubei, Hunan, Guangxi, and Guizhou. The rest included Liaoning, Heilongjiang, Jiangsu, Shandong, Henan, Hubei, Hunan, Guangxi, and Guizhou. Because our study chose occupations in 1989 and 2009 as subjects of occupational mobility, for the fitness of comparison, this study excluded samples from Heilongjiang. Figure 1 shows the eight sampled provinces, which accounts for almost a quarter of the area of China’s territory.

### Research objects

First, we screened the samples based on the following principles. (1) We selected samples from 1989 and 2009 to secure the rationality of the analysis of occupational mobility. (2) We excluded unemployed samples from 1989. (3) We screened the samples and eliminated those who responded “I do not know” to the question “What do you think about your life today?” (4) We selected samples aged 30 to 60. (5) We deleted part of other variables that lacked observation values.

Finally, we obtained 1260 samples. Table 1 describes the sample data. In terms of district distribution, the samples were fairly distributed in the eight provinces. The data showed that the samples had fair representativeness and could show district differences. The rural–urban and gender distributions were also fair.

**Table 1:** Distribution of Samples (N = 1260)

		**Proportion of samples (%)**	**Rural/urban**		**Gender**		**Life satisfaction**	
		**Urban**	**Rural**	**Men**	**Women**	**Others**	**Fair**	
Regional Distribution	Liaoning	167 (13.25)	21	146	108	59	107	60
	Jiangsu	121 (9.60)	23	98	78	43	54	67
	Shandong	139 (11.03)	6	133	107	32	71	68
	Henan	161 (12.78)	20	141	118	43	67	94
	Hubei	198 (15.71)	45	153	132	66	94	104
	Hunan	156 (12.38)	40	116	110	46	75	81
	Guangxi	194 (15.40)	52	142	124	70	140	54
	Guizhou	124 (9.84)	30	94	87	37	70	54

Note: The proportions in the table are parts of the total samples, and thus, the sum of these proportions may not equal the total.

### Variable measurement

#### Dependent variables

Life satisfaction. Many methods can be used to measure the dependent variables. But they can get broadly consistent results in the multivariate analysis [57]. And the questionnaire of CHNS used a single-dimensional model. This study measures life satisfaction by subjective assessment. In the research design, responses to the question “What do you think about your life?” are divided into two categories. The “very good” and “good” answers are taken as good life satisfaction (code 1), whereas the “fair,” “bad,” and “very bad” answers are taken as bad life satisfaction (code 0).

#### Independent variables

(1) Sociology population control variables

In this study, the sociology population control variables were gender, age, marital status, education level, annual income, and Hukou status.

Gender and age were the regular controlled variables. In this study, male and female were coded as 1 and 0, respectively.

We also examined the effect of marital status on life satisfaction. Our study classified marital status into three, namely, single (coded as 1), married (2), and others (including divorced, widowed, and separated; 3).

The degree of education was classified as primary school (coded as 1), secondary school (2), high school (including technical secondary school and vocational school; 3), and university and college (including master’s degree and above; 4).

Income level is measured by the yearly income of the respondent and takes a natural logarithm. Total income includes salary, various bonuses, subsidies, home garden/orchard income, group and family farm income, livestock and poultry farming collective and family income, group and family fishing income, household small handicraft and small home business income, and all other incomes.

Given the Hukou system, this study classified urban and rural residences based on household registration to make this difference clear (urban residence is coded as 1, and rural residence is coded as 2). The urban and rural situation should be measured by residence, and not where people live. Even though they live in urban areas, rural residents have difficulty obtaining the same social/medical security as urban residents. Table 2 shows the methodology of the variables and the basic statistics.

(2) Health status

This is the first core independent variable. Our study chose the question, “In the recent four weeks, have you been sick or hurt? Have you been ill with chronic or acute disease?” These questions represent the health conditions of the respondents. If the answer is NO, the code is 0; otherwise, the code is 1.

(3) Occupational mobility

The most significant independent variable in this research is occupational mobility. The origin refers to the occupation of the respondents in 1989, and the end is the occupation of respondents in 2009 or their last occupation before retirement. Based on the EGP^b^ classification by Goldthorpe, this paper defined the class of respondents. The present paper set up a framework of five occupational categories that represented the occupational status of respondents. Occupational status includes service class (administrative staff, technological staff, and business owners), non-manual worker class (employed clerks in organizations), skilled workers and supervisors (skilled physical workers, low-technological workers, and supervisors of physical workers), and half-skilled/non-skilled workers (non-agricultural and half-technological physical workers), and farmers.

**Table 2:** Statistical description of variables

**Variables**	**Code**	**Value**	**Samples**	**Average**	**Standard deviation**
Gender	0	Female	396	0.6857	0.4644
	1	Male	864		
Age		[32.91,59.99]	1260	48.6123	6.1877
Education level	1	Primary school	351	1.9873	0.7727
	2	Secondary school	607		
	3	High school (including technical secondary school and vocational school)	269		
	4	University and college (including master’s degree or above)	33		
Household registration	0	Rural	1023	0.1881	0.3909
	1	Urban	237		
Marital status	1	Single	10	2.0325	0.2177
	2	Married	1199		
	3	Others (including divorced, widowed, and separated)	51		
Yearly income		[0,288000]	1260	10353.85	24869.17
Occupational status in 1989	1	Service class	50	4.3548	1.1289
	2	Regular non-physical class	83		
	3	Skilled workers	109		
	4	Half-skilled workers	146		
	5	Farmers	872		
Occupational status in 2009	1	Service class	103	4.0492	1.2588
	2	Regular non-physical class	69		
	3	Skilled workers	143		
	4	Half-skilled workers	293		
	5	Farmers	652		
Health status	0	1088	1260	0.1365	0.3435
	1	172			
Life statisfaction	0	Fair (including good, very good)	678	0.4619	0.4987
	1	Others (including middle, bad, very bad)	582		

According to the CHNS, the interviewees who were surveyed on their main occupation and occupational classification in 1989 and 2009 are slightly different. We classified the occupation as follows: service class in high/low-level administrative personnel, professional and technical personnel and large/small business managers included 1 senior professional and technical worker. Managers numbered 3, while administrative officer/managers numbered 8. Routine non-manual class included 2 general professionals, and technical workers numbered 4 among the general office staff. The respondents included 9 soldiers and police officers, and 12 athletes, actors, and performers (in the 1989 survey, Volume No. 14). Skilled workers/supervisors included 6 skilled workers and 10 drivers. Semi-skilled/unskilled workers included 7 non-skilled workers or skilled workers, and 11 service workers (included in the questionnaire in 1989, No. 12). Rural residents included 5 farmers, fishermen, and hunters.

Finally, the 5 × 5 motion table is composed of the original and existing classes. The table indicates the three basic perspectives of occupational mobility. First, when occupational mobility occurs, the main diagonal indicates no occupational mobility, and the code is 0. Non-diagonal indicates the occurrence of occupational mobility, and the code is 1. Second, in terms of the directions of occupational mobility, the diagonal that exhibits downward mobility is coded −1, whereas the one that exhibits upward mobility is coded 1. Third, the distance of occupational mobility indicates the variables between the start and the end. Table 2 shows the methodology of variables and the basic statistics.

### Analysis model (logistic diagonal mobility models)

The earliest analysis model on the impact of occupational mobility and its results was proposed by O. D. Duncan (Clifford and Heath, 1993). In this analysis model, Duncan added the variables of origin class and destination class, and their interaction. The general model of Duncan is demonstrated as

$$Y_{\mathrm{ij}}=X_{i}+X_{j}+X_{i}X_{j}+\epsilon_{\mathrm{ij}}$$

This model is the function that considers the origin and class of a person as dependent variables. *X*_*i*_ and *X*_*i*_ indicate the impact of origin and destination class, respectively, and *X*_*i*_*X*_*i*_ measures their interaction and influence. The most significant challenge of this model is measuring the behaviors and attitudes of migrants. Migrants and non-migrants are measured together, which researchers found hard to distinguish and to determine whether the dependent variable has an impact on occupational mobility. Several scholars have improved this process. In 1981, M. E. Sobar introduced the diagonal mobility model that could distinguish between the effects of mobility and class. This model aided in solving the aforementioned problem. The general form of diagonal mobility model (Abbreviation is DDM) is shown as

$$Y_{\mathrm{ij}}=\mathrm{\eta a}a_{\mathrm{ii}}+\left(1-\eta \right)aa_{\mathrm{jj}}+\epsilon_{\mathrm{ij}}\;\eta \in \left[0,1\right]$$

In this model, *Y*_*ii*_ is the dependent variable, and is the variable in accordance with the normal distribution. *ii* represents the origin class of the respondent. *jj* represents his or her destination class, *aa*_*ii*_ and *aa*_*ii*_ are the general averages of the members of origin and destination class, respectively, who did not drift on the diagonal on the mobile table. *ϵ*_*ij*_ indicates the random error with the average as 0. η and 1-η show the relative weight of origin and destination class, respectively. When η > 0.5, the effect of the origin class is greater than that of the destination class in occupational mobility. When η < 0.5, the effect of the destination class is greater than the origin class. DMM considered the difference between the effects of the origin and the destination, but did not consider the existence of mobility effect. The standard model completely excluded the drift effect and examined whether the drift effect existed by adding drift variables. At the same time, the standard model added controlled variables in the standard model to form a total model

$$\begin{array}{l} Y_{\mathrm{ij}}=\mathrm{\eta a}a_{\mathrm{ii}}+\left(1-\eta \right)aa_{\mathrm{jj}}\\ \;+\sum_{\omega =1}^{\omega }\beta_{\omega }X_{\mathrm{ij\omega}}+\sum_{\varpi =1}^{\varpi }\beta_{\varpi }Z_{\mathrm{ij\varpi}}+\epsilon_{\mathrm{ij}} \end{array}$$

where *X*_*iiω*_ and *Z*_*iiϖ*_ are the drift (including the three factors of whether the drift exists, direction, and distance of the drift) and controlled (including the five factors of gender, age, household registration, education level, and income level) variables. Correspondingly, *β*_*ω*_ and *β*_*ϖ*_ are the variable index of drift and controlled variables, respectively. In this study, the logistic diagonal mobility models are applied because life satisfaction is a classified variable.

## Results

### Descriptive analysis of types of occupational mobility and life satisfaction

We built a 5 × 5 occupational mobility table and calculated the life satisfaction in each single table. The results are presented in Table 3, which also showed the differences among life satisfaction.

**Table 3:** Descriptive analysis of occupational mobility and life satisfaction

	**Service class**	**Regular non-physical class**	**Skilled workers**	**Half-skilled workers**	**Farmers**	**Total**
Service class	60.61	44.44	100.00	50.00	33.33	46.67
	(33)	(9)	(1)	(4)	(3)	(50)
Regular non-physical class	72.73	50.00	44.44	50.00	50.00	49.46
	(22)	(26)	(9)	(16)	(10)	(83)
Skilled workers	80.00	78.57	52.00	46.34	47.37	52.17
	(10)	(14)	(25)	(41)	(19)	(109)
Half-skilled workers	91.67	53.33	38.89	53.23	42.86	47.47
	(12)	(15)	(36)	(62)	(21)	(146)
Farmers	50.00	60.00	44.44	36.47	43.91	35.29
	(26)	(5)	(72)	(170)	(599)	(872)
Total	66.02	56.52	44.76	42.32	44.02	46.19
	(103)	(69)	(143)	(293)	(652)	(1260)

#### Mixed effects analysis

A clear stage of life satisfaction existed in the perspective of the relationship between current occupation and life satisfaction. The percentage of the service class who thought they have good life satisfaction is 66.02%, and those of the regular physical class, skilled workers, half-skilled workers, and farmers are 56.52%, 44.76%, 42.32%, and 44.02%, respectively. The maximum stage among the different stages is 66.02% to 44.02% = 22.00%. In the perspective of origin class (the occupational status in 1989) and its relationship with the life satisfaction, the difference still exists but the stage difference is not as significant as the current occupation. As 52.17% is the highest life satisfaction, as rated by a respondent who is a skilled worker in 1989, the difference between each stage is 16.88% (52.17% to 35.29%). The drift effects are mixed when considering the impact of origin and destination class on life satisfaction only.

#### Excluding the effects of occupational mobility

From the position of the diagonal in Table 3, except for occupational mobility effect (only concerning the stage of non-migrant life satisfaction in different social classes), the percentages of Chinese residents of different occupations who have good life satisfaction in the service class, regular non-physical class, skilled worker class, half-skilled worker class, and farmers are 60.61%, 50.00, 52.00%, 53.23%, and 43.91%, respectively. The difference between each stage is 16.70% (60.61% to 43.91%). This result indicates that if no occupational mobility occurs, then the maximum difference between each stage of the life satisfaction is 16.70%. Thus, we preliminarily judged that occupational mobility has a mixed effect on life satisfaction. Farmers had the lowest life satisfaction on the total table for both 1989 and 2009. This result indicates that, to a certain degree, occupation affects life satisfaction.

### Logistic diagonal mobility models

This study applied multi-variable statistical analysis in investigating the relationship between occupational mobility and life satisfaction of Chinese residents of different occupations through the logistic DDMs to eliminate the impact of other variables on the relationship between occupational mobility and life satisfaction. Model 1 is the basic model, which consisted of the standard model and controlled variables, and excluded the drift variables. Model 2 is the total model, which consisted of all concerned variables in this study. Model 3 is the screening model, which excluded the insignificant drift effect index in Model 2. Table 4 shows the results.

**Table 4:** **Logistic DDMs on the relationship between** occupational **mobility and life satisfaction**

**Variable**	**Model 1**		**Model 2**		**Model 3**	
	**Standard model**		**Total model**		**Screening Model**	
	**Coefficients**	**Standard deviation**	**Coefficients**	**Standard deviation**	**Coefficients**	**Standard deviation**
Origin effect	0.5180	1.1867	0.7410	1.1865	0.6256	1.1854
Intercept	−2.4393	0.8735^***^	−1.2902	0.7899	−1.5637	0.8083^*^
Hukou (Rural = 0)	−0.2672	0.1563**	−0.2681	0.1562*	−0.2244	0.1558
Gender (Female = 0)	−0.0891	0.1276	−0.1077	0.1276	−0.0900	0.1275
Age	0.0181	0.0096^*^	0.0131	0.0096	0.0139	0.0096
Education level						
Secondary school	0.1636	0.0713^**^	0.1326	0.0711^*^	0.1456	0.0711^**^
High school	0.2476	0.0575^***^	0.2291	0.0574^***^	0.2361	0.0573^***^
University and college	0.2146	0.0282^**^	0.1601	0.0965^*^	0.1855	0.0969^*^
Marital status						
Married	0.4003	0.3694	−0.0184	0.3263	0.0759	0.3308
Single, widow	−0.1296	0.2713	−0.4756	0.2488^*^	−0.3796	0.2497
Natural logarithm	0.0024	0.0056	0.0033	0.0056	0.0024	0.0055
Health status (Health = 0)			−0.4877	0.1731^***^	−0.4206	0.1720^**^
Whether occupational mobility happened (Yes = 1)			−0.0895	0.0851		
Direction of occupational mobility			−0.1028	0.0026^***^	−0.0965	0.0019^***^
Distance of mobility			0.1980	0.0103^***^	0.2355	0.0114^***^
AIC value	1717.2	1722.1	1719.7			
BIC value	1784.0	1804.3	1796.8			
Samples	1260	1260	1260			

Note: ^**·**^ means the significance of 10%; * means the significance of 5%; ** means the significance of 1%; *** means the significance of 0.1%.

Table 4 shows that the origin effect η of the three models is greater than 0.5, which indicates that the origin effect is greater than the destination effect. The effect of the origin class on life satisfaction is greater than that of the destination class.

#### Control variables

The controlled variables in the model are household registration, gender, age, education level, marital status, and yearly income (in the form of natural logarithm). In Model 1, household registration, age, and education level have a significant effect on life satisfaction of Chinese residents of different occupations. However, only residence, education, and marital status have significant effects in Model 2, and only education has a significant effect in Model 3.

(1) From the perspective of household registration, rural samples had better life satisfaction than urban samples.

Two reasons may explain this finding. ***First,*** the living environment, such as air and water, in urban areas is worse than that in rural areas. China’s rapid economic development largely came at the cost of environmental damage. Environmental problems have become a global problem that greatly reduces the quality of life. Urban environmental quality, including water and air pollution, affects the health of rural and urban residents [53]. ***Second,*** with urban expansion, the urban population is facing greater inflationary pressures. The work and life pressures of urban residents are relatively greater than those of rural residents. A survey of the life satisfaction of Beijing residents found that duration of stay in Beijing, and ownership class position negatively affect residents’ life satisfaction. Residents who have stayed in Beijing for a long time have lower life satisfaction because they might not have been able to adapt to the modernization of the city [58].

(2) From the perspective of age, older people have higher life satisfaction,

This finding is consistent with previous findings that life satisfaction increases with age [58]. This increase is most likely due to the fact that older persons have fewer demands and are easily satisfied, whereas younger people have higher self-fulfilling requirements and become unhappy with minor problems, which decreases their life satisfaction. In addition, from an objective point of view, the fast pace of modern life and strong competition further decreases their life satisfaction.

(3) Based on the results, we determined that high school graduates have better life satisfaction than university graduates, and the latter have better life satisfaction than secondary school graduates.

A Swedish scholar studied the likelihood of gross life satisfaction among 18- to 64-year-old Swedes. He found that university educated people were less satisfied when meeting with friends and acquaintances compared with people who have lower education, which may be caused by the fact that university educated people do not invest in emotional and leisure time [59]. Such a lack of investment in personal time may be due to a higher level of needs, such as self-esteem and self-fulfillment (Maslow’s hierarchy of needs) when one is more highly educated [60]. Hence, the respondent may subjectively think that he or she has poor life satisfaction because of work and life pressures.

(4) From the perspective of marriage, divorced or widowed individuals have poor life satisfaction.

This conclusion is consistent with general perceptions. Divorced or widowed people are unattended and tend to live alone. In addition, they lack an important confidante with whom they can share their worries, which will reduce life satisfaction. Having a partner can significantly ease their emotional burden and stress, hence improving their life satisfaction [59,61].

#### Analysis variables

From the perspective of the analysis of controlled variables, health conditions, direction, and distance of occupational mobility significantly affected life satisfaction of Chinese residents of different occupations.

(1) First, from the perspective of health status, respondents who have not been sick or injured had better life satisfaction than those who had been sick or injured.

From an objective point of view, poor health leads to inconvenient life mobility and economic burden, which reduces the life satisfaction of respondents. Studies found that when the elderly have poor self-perceived oral health status, they would report a poorer quality of life [62]; oral health is related to general health than overall well-being [63]. According to a Swedish scholar, poor health contributes to serious sexual dysfunction, which reduces sexual satisfaction [59]. From a subjective perspective, a negative view of one’s health will negatively affect one’s psychological conditions, which leads a person to feel that his or her life is unhappy and therefore rate his or her life satisfaction as “poor.”

(2) Second, from the perspective of occupational mobility direction, the coefficients of occupational mobility in the models are less than 0, which means that upward mobility negatively affects life satisfaction.

Though Occupational upward mobility is success oriented and increases one’s purchasing power, and income and occupational status are a crucial factor in life satisfaction [64]. However, meeting economic needs does not signify an improved overall well-being. ***First,*** psychologically, each person has a need to fit in with a group and to communicate with others. After entering a new environment, they need to adapt to its rules and culture. However, if they are unable to adapt, the anxiety and isolation they experience would make them feel as though they have lost their dignity and social status. Isolation from a mainstream group or self-abasement may make fitting into a new environment difficult for a person, which might result in poor life satisfaction.

***Second,*** with upward mobility, work and life pressures increase because the change may require them to be part of a more intense environment as well as drive them to cultivate higher levels of need. This condition could decrease life satisfaction. A Chinese scholar studied the reason for the unhappiness of rural–urban migrants and found that they had false expectations of the future. Migrant conditions and high expectations to succeed contributed to unhappiness [65].

***Third,*** before upward mobility occurs, one may have a high expectation of one’s future, and the gap between expectations and reality will decrease life satisfaction. In fact, work pressure negatively affects professional well-being [66]. If people are unable to cope with the pressure from their new career, then their life satisfaction will be reduced.

(3) Third, when analyzing mobility distance in Models 2 and 3, a greater distance indicates better life satisfaction.

Upward mobility requires one to adapt to a new environment as well as presents new pressures in life. Upward migration sometimes means the negative effects. A scholar found that upward migration decreased the fertility [67]. Occupational mobility may have a very subtle effect on a person’s mental state. Some scholars used the self-concordance model to explore how core self-evaluations affect work and life satisfaction, and found that core self-evaluations can motivate the pursuit of goals [68,69]. Thus, the psychological and financial incentive effects exist as one move upward in his or her career. Upward mobility leads to a higher income and better living conditions, although the aforementioned problems still exist. Thus, greater occupational mobility distance could lead to better life satisfaction of Chinese residents of different occupations.

## Conclusions and discussions

This paper analyzes the life satisfaction of the Chinese residents of different occupations from the perspective of occupational mobility, with other factors taken into consideration, including household registration, gender, age, education level, marital status, annual income, health status, and occupational flow direction and distance. The results of this study show that occupational mobility life satisfaction has a mixed effect and that people’s occupation affects their life satisfaction. Overall, in the three models, the origin effect η is greater than 0.5, which is greater than the destination effect. This finding indicates that the class from which the respondents came has a greater effect on their life satisfaction than the origin impact. This finding is detailed as follows:

1. Among the control variables, household registration, education, age, and marital status significantly affect life satisfaction of Chinese residents of different occupations. Hypothesis 1 is supported.

(1) For model classification

(a) In Model 1, household registration, age, education level and other factors significantly affect life satisfaction;

(a) In Model 2, household registration, marital status, and education level significantly affect life satisfaction.

(a) In Model 3, only education is significant.

(1) For variable classification

(a) In Models 1 and 2, the effect of household registration status on life satisfaction was statistically very significant. Household registration is closely related to life satisfaction, and life satisfaction in urban areas is worse than that in rural areas.

(a) From the age perspective, age is significant only in Models 1 and 2. Older persons have higher life satisfaction.

(a) From the education perspective, the education variables in Models 2 and 3 are more significant. High school graduates and respondents with an equivalent education level have greater life satisfaction than college graduates, while the latter have higher life satisfaction than respondents with junior high school education.

(a) From the perspective of marital status, marital status is only significant in Models 1 and 2. Being divorced or widowed leads to poor life satisfaction.

These findings show that the life satisfaction of Chinese residents with different occupations varies with different sociological population control variables. Overall, its distribution characteristic is roughly consistent with the life satisfaction of residents from other countries, but also has its own distinct characteristics. Environment and stress is worse in urban areas than in rural areas, which affects urban residents’ attitudes. As people grow older, their need for self-realization decreases, which can enhance their life satisfaction. Similarly, highly educated people are more likely to work hard to achieve more. They have higher demands, which increase work and life pressures, thereby reducing their life satisfaction. For the marital status variables, the results are consistent with commonly held views, which is that having a partner can improve life satisfaction.

2. The analysis of controlled variables indicates that health conditions, direction, and distance of occupational mobility are related to life satisfaction of Chinese residents of different occupations, which is a major concern. Health conditions, direction, and distance of occupational mobility had a statistically significant effect on life satisfaction of Chinese residents of different occupations.

(1) From the perspective of health conditions, respondents who have not been sick or injured had better life satisfaction than those who had been sick or injured. Hypothesis 2 is supported.

This finding is consistent with our objective understanding that illness greatly inconveniences everyday life. Medical costs are an economic burden, and worries about the future can affect a person’s psychological state, thereby reducing his or her sense of happiness. Particularly in China, the lives of residents with different occupations basically revolve around work. Illness hinders their normal working life, which imposes a major financial burden. In view of this situation, improving people’s health or eliminating their concerns and fears is an important way of enhancing life satisfaction.

(1) In our study, the direction of occupational mobility is a key factor that influences life satisfaction and cannot be ignored. From the perspective of occupational mobility direction, we can conclude that upward mobility has a significantly negative effect on life satisfaction.

(1) From the perspective of distance, when analyzing mobility distance in Models 2 and 3, a greater distance indicates better life satisfaction. Hypothesis 3 is supported.

Thus, by measuring the life satisfaction of Chinese residents of different occupations and its links to occupational mobility, we can conclude that occupational mobility for life satisfaction had a mixed effect on occupational mobility, that is, life satisfaction will have a positive or negative effect. The process is complex, which means that upward occupational mobility does not necessarily increase life satisfaction and that downward occupational mobility does not diminish life satisfaction. This situation may be explained in two ways: upward mobility indicates a good ability to adapt to a new environment and to deal with new pressures. Inability to adapt to a new environment will confuse people, thereby reducing their life satisfaction.

In the background of the universal differentiation of Chinese occupations, this matter is a new issue. Previously, scholars studied obstacles encountered by individuals prior to experiencing occupational mobility to address the reason for people’s decreased happiness despite the country’s economic growth [70]. Eastern European countries also face the issue of declining public life satisfaction, which resulted from lower salaries and employment levels, the increased gap between wages and employment, and disrupted family life [71]. In China, reduced life satisfaction may be attributed to income inequality [70] despite the greater role of market factors in employment configuration assignment since the opening-up reform. Institutional constraints and structural constraints of the rational flow of professions still exist, which results in social inequalities [72]. A Chinese scholar found that intergenerational turnover of farmers is quite high, and cities also have “open” social mobility based on a national sample survey data of contemporary Chinese life history and social change in 1996. He believes that this model is caused by the unique household registration system in China, which ensures rural off-farm employment and allows farmers’ children who did not change hukou to continue farming. The system only allows rural people with high education to have access to urban hukou [73]. The hukou system eases the relationship between job satisfaction and life subjective well-being [74]. Chinese residents of different occupations suffered unequal treatment at the starting point of occupational mobility have been at a disadvantage when competing for jobs, but there are still many Chinese unreasonable policies and institutions to further strengthen these disadvantages in the process of occupational mobility. And this is the biggest social inequity China is currently faced with. Many other inequalities are derived or brought from this inequality [72]. Changing system inequalities in the process of occupational mobility can reduce social inequality (including the income gap), thus removing barriers of occupational mobility in different areas.

We studied people’s psychological condition after occupational mobility occurred. Issues such as problems with government, individual improvement, individual rights, and other aspects of poor liquidity were observed in occupational mobility of residents in China. These problems are due not only to the restrictions of the social system, but also the lack of capital and social capital of individual migrants. Human capital directly affects occupational mobility decisions. In the process of migrants’ occupational mobility, human capital is more important than social capital [75]. After the occurrence of occupational mobility, social adaptation becomes an important factor that affects life satisfaction of Chinese residents of different occupations.

To address the findings, this study presents a number of recommendations. ***First,*** companies should provide corresponding job training and psychological guidance for new employees. A harmonious and friendly working atmosphere should be created to allow new employees to integrate quickly and reduce feelings of loneliness and exclusion. ***Second,*** effective interventions for mental psychological problems should also be carried out for people with different occupational mobilities. Such interventions can include assisting people to find effective ways to ease work and life pressures in the city to help them adapt to urban living and improve their problem solving skills.

This study has some limitations. ***First,*** the data were obtained from a nationwide CHNS survey. Only a single question is used to measure overall life satisfaction among individuals with different occupations. This approach does not allow us to develop a more comprehensive understanding of which area of life is the residents dissatisfied with [76]. ***Second,*** the study does not discuss in detail the occupational mobility of different occupations. Future research can target inflows and outflows factors of a particular occupation and conduct more detailed analysis that combines changes in life satisfaction. ***Third,*** we should conduct a long-term sustained study of life satisfaction of residents of different occupations in China. A comparative tracking study can be performed by using updated CHNS data.

## Endnotes

^a^The CHNS website, URL is available: http://www.cpc.unc.edu/projects/china.

^b^The six occupational stages of EGP are service class, regular non-physical, self-employed and small property owner, skilled workers, unskilled workers, and farmers. Based on the CHNS survey, this paper excluded self-employed and small property owner.

## Competing interests

The authors declare that they have no competing interests.

## Authors’ contributions

YL wrote and revised the manuscript, was responsible for the design of the study, and performed the statistical analysis. PL participated in the design and writing of the study. Both authors read and approved the final manuscript.
